# Notes on Ophthalmia Neonatorum

**Published:** 1909-07-31

**Authors:** Philip A. Harry


### Ophthalmology.

### NOTES ON OPHTHALMIA NEONATORUM.

By PHILIP A. HARRY, M.D., D.P.H.

The statistics of the Ophthalmic Department of this hospital* for the last ten years show practically the same number of cases each year, with a slight tendency to increase. The average number lies between 20 and 25. The disease has a seasonal incidence, the greater number of cases occurring in spring and summer. In autumn and winter, especially in cold years, the cases are less severe and the complications not so numerous.

Only complicated cases are admitted, chiefly on account of intense œdema of the lids, purulent nasal discharge, and corneal infections. The corneal lesions varied from a slight haze to a total necrosis. In one case the sloughing cornea presented a peculiar honeycomb appearance. Dry xerotic cornea has also been noticed. Slight and uncomplicated cases of more than three or four weeks' duration are not admitted, but are treated daily as out-patients.

For the year ending June 30, 1909, there were 29 admissions; 21 of these patients had not been attended by a medical practitioner.

First and second children were those most often affected. In two cases, the tenth and eighth children were attacked, but in both instances inquiry showed that they were first-borns of a second marriage. With very few exceptions it was found that the mother suffered from a purulent vaginal discharge for some time before the birth of the child; the importance attached to this by the Ophthalmia Neonatorum Committee is certainly not uncalled for.

The following case emphasises this point: Mrs. C., aged 32, married twelve months previously, brought a male child for advice and treatment. The history was that he was 14 days old and a twin. The second child was born 11½ hours after the first; no instruments were used; the head of the first child was born some time before the rest of the body. On the third day it was noticed that his lids were swollen and a discharge was coming from the eyes. The medical attendant saw the child once and recommended hourly bathings with boracic lotion; no drops were used. A fully trained nurse was also in attendance, the mother was certain that immediately after the birth the child's eyes were carefully wiped with separate pieces of cotton-wool and the head bathed first from a separate bath than that in which the rest of his body was washed. In answer to a direct question, she replied that she had a discharge for a long time. Examination of the child showed that he was a poorly developed emaciated infant; the lids were red and the margins covered with a thick yellow discharge. Careful separation of the lids with a small-sized Desmarre's retractor showed also that the lower parts of both corneæ were ulcerated. The child was admitted. The second child did not develop the disease. This interesting case emphasises the fact that prophylaxis to be successful must be directed towards purulent discharges in the mother.

* The Leeds General Infirmary.

Complicated labours were very rare among the 29 admissions, as 73 per cent. were attended by midwives alone. In the majority of these cases no history could be obtained of any prophylactic measures having been taken to prevent infection of the child's eyes, not even the precaution of cleaning the head and face before the body.

The cases admitted fall, roughly, into three classes—early cases brought by the friends or neighbours within the first week of onset; late cases in the third or fourth week brought by the mother; and an intermediate class, infants from 10 to 14 days old, also brought up by the mothers.

Three-fourths of the cases fall in this latter group; they are usually severe because the mother has waited until she is able to get about before bringing up the child. The treatment at home has been most inefficient, consisting very often only of the "dropping in" of a little boracic lotion occasionally; corneal complications are consequently present in more than 50 per cent., and the stay in hospital averages three to four weeks. The early cases are usually discharged cured at the end of a week; the late cases take a fortnight, more or less. The mothers are always strongly advised to come into hospital with their infants, especially those of the intermediate group.

Infants admitted alone rapidly lose ground; they become pale and emaciated, their digestive functions are easily impaired, and the power of resistance decreases. In the last five years there have been six deaths among the children of this class admitted without their mothers. It is frequently found, too, that after their return home they thrive badly owing to convulsions and intestinal troubles.

Babies of over four weeks are rarely admitted, except when frequently repeated relapses threaten further complications. Microscopic examination shows that these are nearly always mixed infections. On or before admission an examination is made of the discharge from the eyes, and from the nose should there be a nasal discharge as well. The gram-negative diplococcus of Neisser is practically always found in the film. A culture of the organism can also be made for the purpose of producing a vaccine.

As soon as possible, as part of the routine treatment, the external canthi are slit for about a quarter of an inch, to facilitate the proper cleansing of the conjunctival sac. The nurse whose sole duty it is to look after these cases carefully washes, every half hour, the everted lids and fornices. The lotions in chief use are glycothymolin, 1 in 5; boracic acid, ℥ij to the pint; potassium permanganate, 1 in 5,000. Alum lotions are said to dissolve the cement substance of the cornea. Mercurial lotions also are not commonly used, on account of their deleterious effect on the cornea, as well as certain other bad results, to be mentioned later. Within the first 24 or 36 hours, when the discharge is thin and serous, the lotion is kept cold by means of lumps of ice. Results show that these early cases respond very readily to prompt and careful treatment. After the third day the lotion is used at body temperature; the discharge has now become purulent.

The morning after the canthi have been slit the mucous membrane of the lids is painted with a strong solution of silver nitrate, gr. xx to the ounce. This causes a temporary increase in the œdema of the lids and a superficial slough, which takes some days to separate. Argyrol drops, 20 per cent, are instilled every four hours. In a few cases 1 c.c. of a vaccine, containing 50 to 75 million gonococci, has been injected into the buttock, without definite benefit. No bad results were, however, noted. There was never even a rise of temperature.

The discharge markedly decreases by the fifth or sixth day; notwithstanding this, treatment should be persevered with for ten days altogether. Successful results depend on frequent bathing of the conjunctival sac, and an intelligent variation of the lotions according to the progress of the case. Instruments such as the flushing retractor and speculum are not recommended for douching. A 1 per cent. solution of atropine sulphate is used only when corneal complications are present: the solution is preferable to the ointment. Special attention is, of course, paid to the feeding of the infant.

It is not enough for the family physician to prescribe a lotion and see the child occasionally. Efforts to combat the disease must be thorough and unceasing. More often than not one finds after inquiry that only the outer surfaces of the lids, or at most the lid margins, have been mopped with the lotion, while the conjunctival sac and fornices are filled with purulent discharge. On the other hand, forcible separation of the lids by an inexperienced person may cause a corneal abrasion, which will become the seat of infection. It is almost impossible to evert the œdematous lids and reach the fornices without slitting their external angles. The operation is easily and quickly performed, and can be done in the wards without an anæsthetic. The infant's head is steadied by the nurse or between the operator's knee; with a sharp Graefe knife a small incision is made down to and through the orbicularis muscle, thus separating the lids and their outer angles. The incision is made from without inwards, the point of the knife being directed towards the temple. A pair of scissors is not recommended, because, even if very sharp, it causes more pain than a Graefe knife, and there is always the risk if the points are not very blunt of scratching or penetrating the cornea. The temporary disablement of the orbicularis, as well as the slight increase in the palpebral fissure render eversion of the lids an easy matter.

Hæmorrhage is never alarming; it has a beneficial effect on the œdema; pads may be applied if it does not stop quickly.

Artery forceps are never needed, except for older children with purulent ophthalmia. No scar is left afterwards, and the wound is quite healed at the end of a week; it never becomes infected. Care should be taken when painting the lids with silver nitrate not to touch the wound, as it is very painful, and a persistent slough is apt to form. A 5 per cent. solution is preferred, because only one application is necessary, as the superficial slough takes some days to disappear; weaker solutions require to be used more frequently. The effect is kept up by the argyrol, which is certainly superior to all the silver preparations of its kind. It is not advisable to dust powders into the eye, not even if the particles are very fine.

Mercurial lotions have no advantage over the milder lotions, seeing that a cleansing rather than a sterilising effect is desired. Pallor and profuse salivation, which stopped after biniodide lotion was discontinued, were noticed in more than one case: and several years ago, when perchloride lotion was used stronger than at present, two infants died suddenly from acute gastro-enteritis a few days after admission. It is advisable, too, when the mothers have to do the eyes, that they be given a bland non-poisonous lotion. The disadvantage of the half-hourly treatment as carried out in hospital is the consequent disturbance of the infant's rest. It does not apply to cases treated at home, for obvious reasons. After the first 24 or 36 hours the eyes are bathed hourly or two-hourly only, during the night and as gently as possible.

In the very early cases, when the babies are on ice-cold lotion, care must be taken to keep them warm, especially after the eyes have been bathed.

Other points to be remembered are, that the child should lie on the side corresponding to the worst eye, to prevent the discharge or lotion trickling into the better eye, which should always be done first; and that in case the nurse has more than one child to look after, the same principle should be carried out. It is very rare for a child to be admitted with only one eye affected, although it is usual for the severity to be unequal. Attempts to seal up the good eye are not always effectual.

The mother and attendants should be warned about the possibilities of infecting their own eyes: it is, however, remarkable that, notwithstanding the close proximity of mother and child, one has never seen a gonorrhœal infection of the mother's eyes, whereas other conjunctival infections are readily transmitted; immunisation of the mother possibly has something to do with it. The child must be carefully protected from draughts or sudden changes of temperature, as relapses are not uncommon, and they retard progress.

With regard to after treatment, the mothers are told to continue the treatment at home three or four times a day for several weeks. Cases of corneal opacities are given an ointment of thiosinamin and dionin in the proportion of 2 and 4 per cent. This is used for many months, varied occasionally with the yellow oxide of mercury (1 per cent.).

Staphylomatous globes should be excised. Some of the complicated cases were written for twelve or eighteen months afterwards. They nearly all developed nystagmus or strabismus; this was invariably the case when the cornea had perforated, as evidenced by the anterior synechia and anterior polar cataracts.

